# Supplementary figures and images for: The mechanism underlying B-cell developmental dysfunction in Kawasaki disease based on single-cell transcriptomic sequencing
Source: Front Immunol. 2024 Oct 23;15:1438640. doi: 10.3389/fimmu.2024.1438640 (PMC11537935; doi:10.3389/fimmu.2024.1438640)

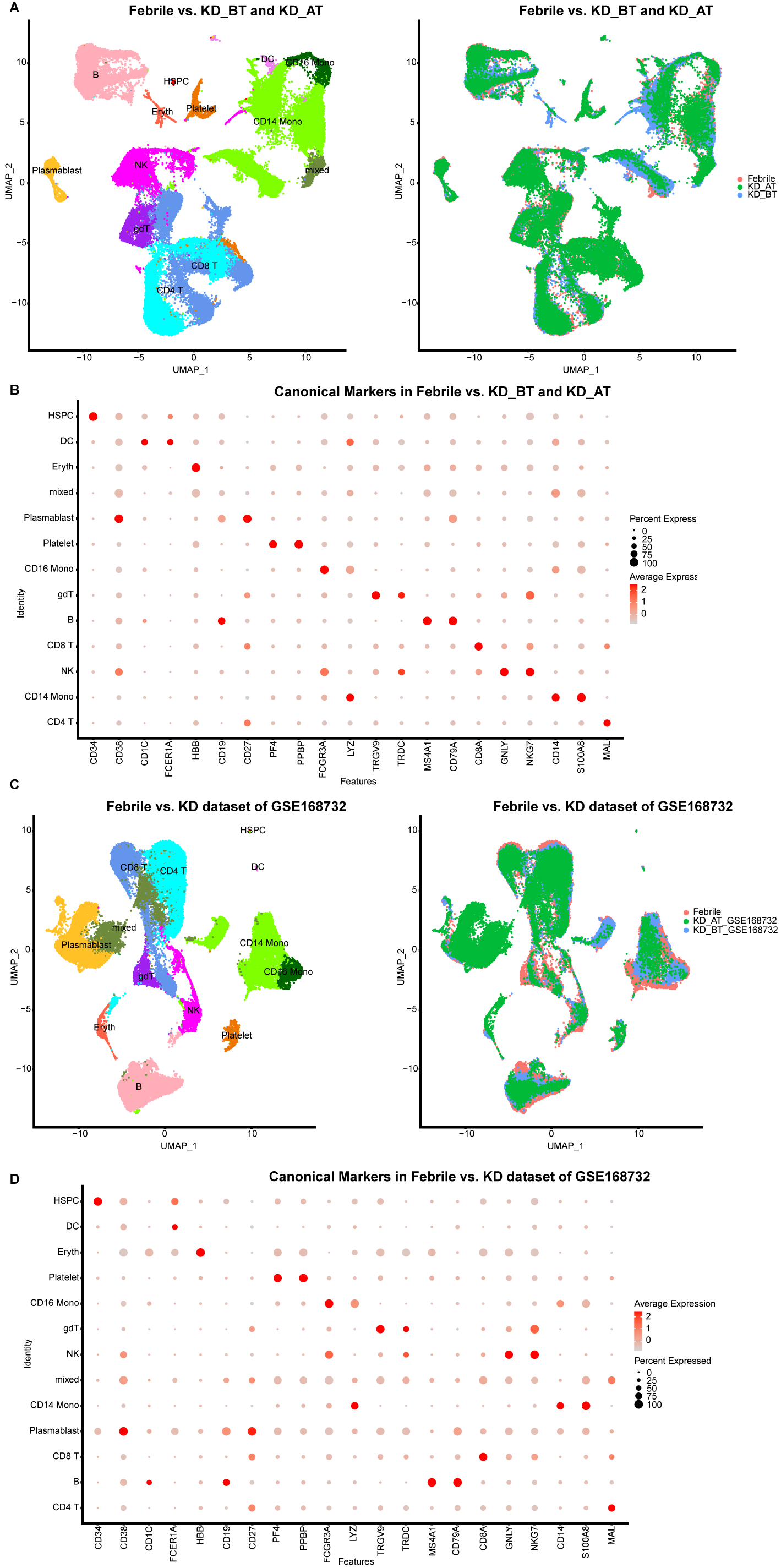

Supplement: Supplementary Figure S1 — Integrated single-cell profiling of PBMCs for our dataset and GSE168732 dataset. (A) Integrated single-cell profiling of PBMCs for our dataset. (B) Expression of canonical gene markers for each cell type in our dataset based on integration analysis. (C) Integrated single-cell profiling of PBMCs for GSE168732 dataset. (D) Expression of canonical gene markers for each cell type in GSE168732 dataset based on integration analysis. The inferred cell types are marked with different colors. [file Image1.tif]

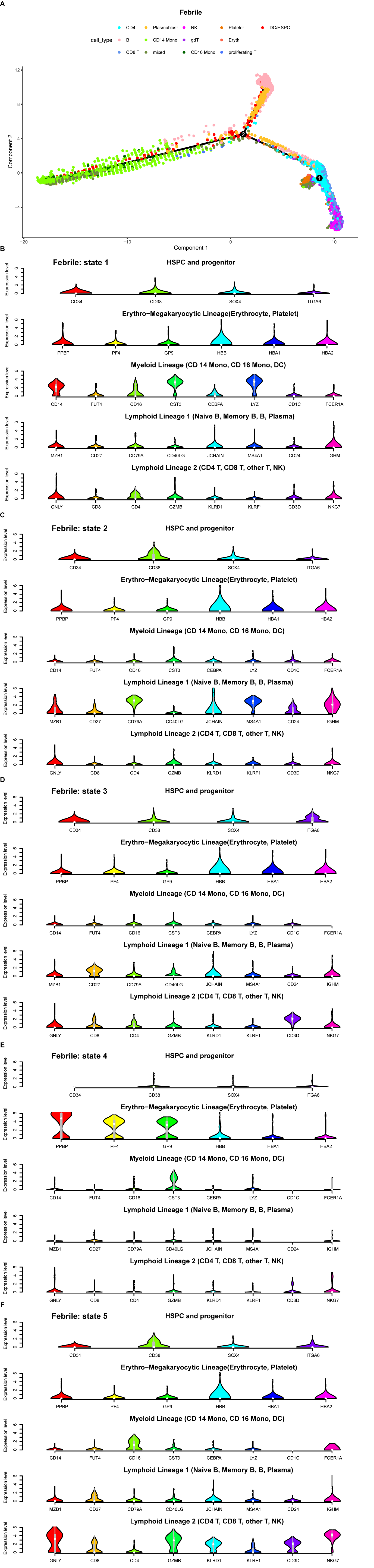

Supplement: Supplementary Figure S2 — Pseudo-time analysis of cell developmental trajectory in febrile patients of our dataset. (A) The differentiation trajectory of all cells in febrile patients by cell types in our dataset. (B) The canonical markers of five cell lineages for state 1 in febrile patients in our dataset. (C) The canonical markers of five cell lineages for state 2 in febrile patients in our dataset. (D) The canonical markers of five cell lineages for state 3 in febrile patients in our dataset. (E) The canonical markers of five cell lineages for state 4 in febrile patients in our dataset. (F) The canonical markers of five cell lineages for state 5 in febrile patients in our dataset. [file Image2.tif]

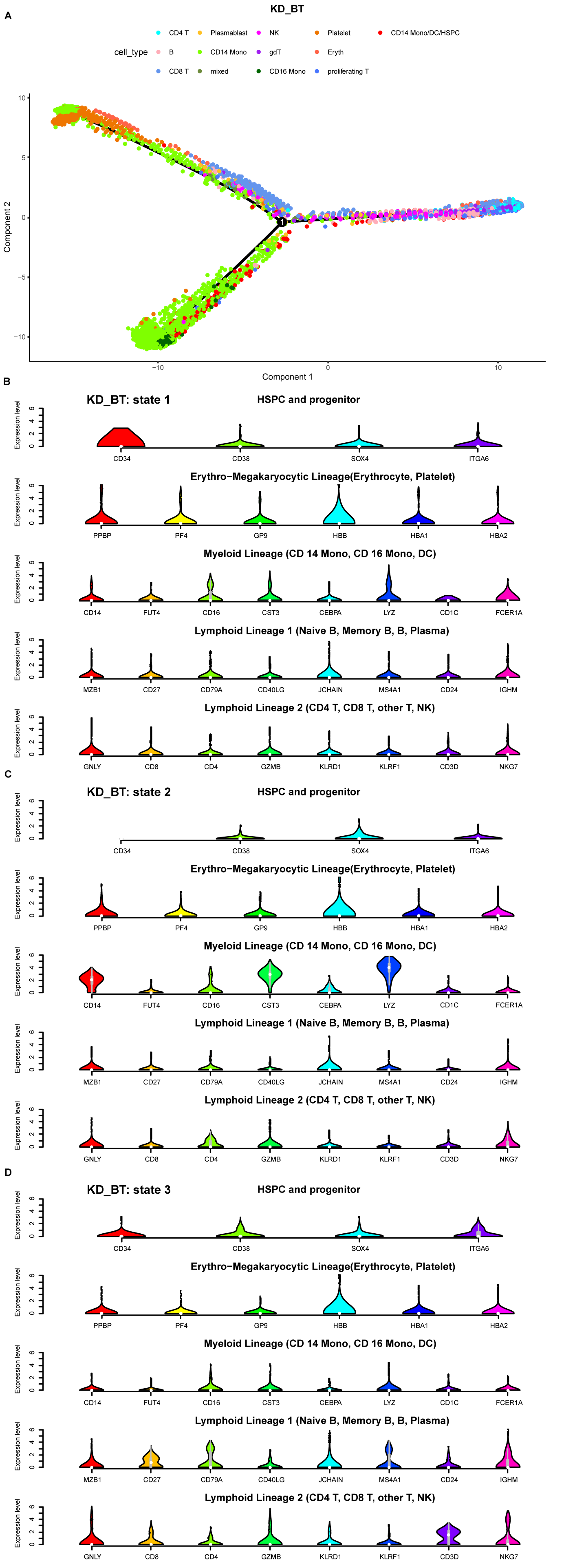

Supplement: Supplementary Figure S3 — Pseudo-time analysis of cell developmental trajectory in KD patients before treatment of our dataset. (A) The differentiation trajectory of all cells in KD patients before treatment by cell types in our dataset. (B) The canonical markers of five cell lineages for state 1 in KD patients before treatment in our dataset. (C) The canonical markers of five cell lineages for state 2 in KD patients before treatment in our dataset. (D) The canonical markers of five cell lineages for state 3 in KD patients before treatment in our dataset. [file Image3.tif]

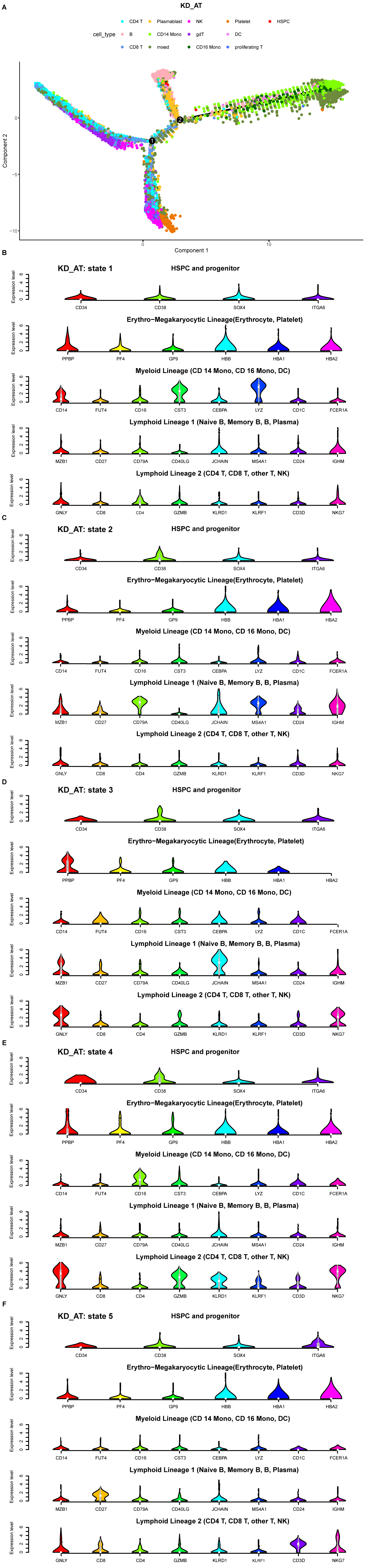

Supplement: Supplementary Figure S4 — Pseudo-time analysis of cell developmental trajectory in KD patients after treatment of our dataset. (A) The differentiation trajectory of all cells in KD patients after treatment by cell types in our dataset. (B) The canonical markers of five cell lineages for state 1 in KD patients after treatment in our dataset. (C) The canonical markers of five cell lineages for state 2 in KD patients after treatment in our dataset. (D) The canonical markers of five cell lineages for state 3 in KD patients after treatment in our dataset. (E) The canonical markers of five cell lineages for state 4 in KD patients after treatment in our dataset. (F) The canonical markers of five cell lineages for state 5 in KD patients after treatment in our dataset. [file Image4.tif]

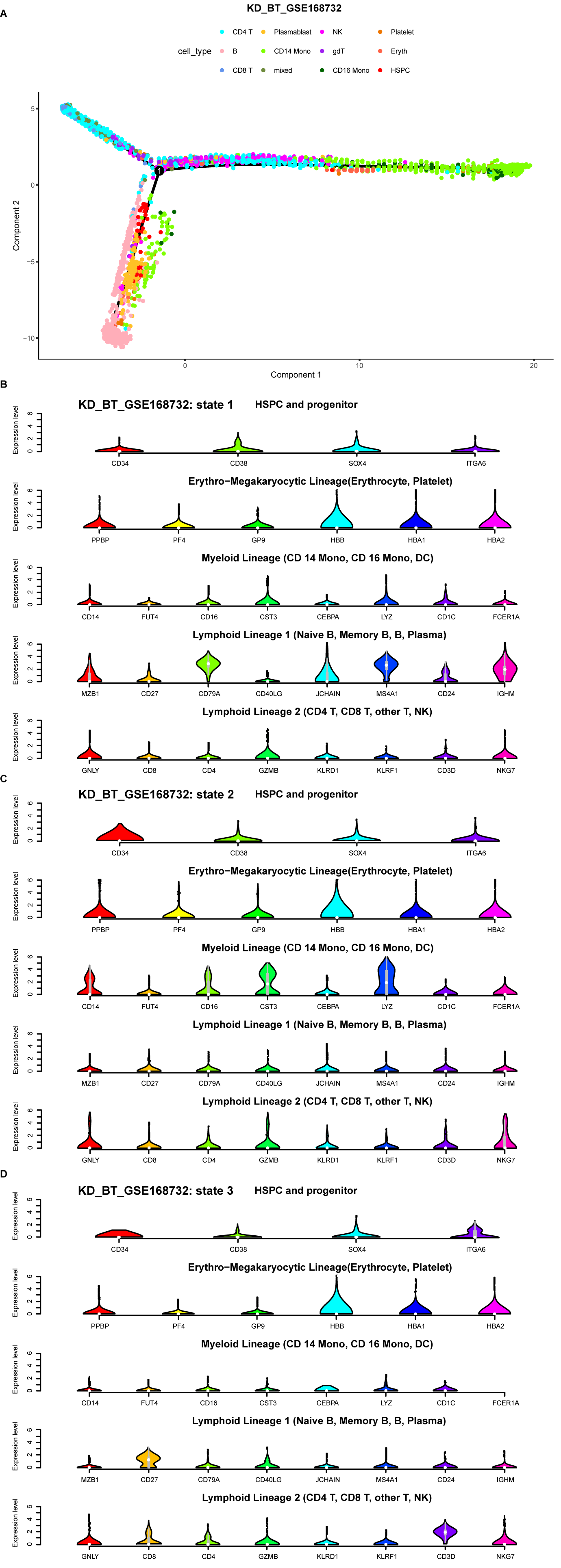

Supplement: Supplementary Figure S5 — Pseudo-time analysis of cell developmental trajectory in KD patients before treatment of GSE168732 dataset. (A) The differentiation trajectory of all cells in KD patients before treatment by cell types in GSE168732 dataset. (B) The canonical markers of five cell lineages for state 1 in KD patients before treatment in GSE168732 dataset. (C) The canonical markers of five cell lineages for state 2 in KD patients before treatment in GSE168732 dataset. (D) The canonical markers of five cell lineages for state 3 in KD patients before treatment in GSE168732 dataset. [file Image5.tif]

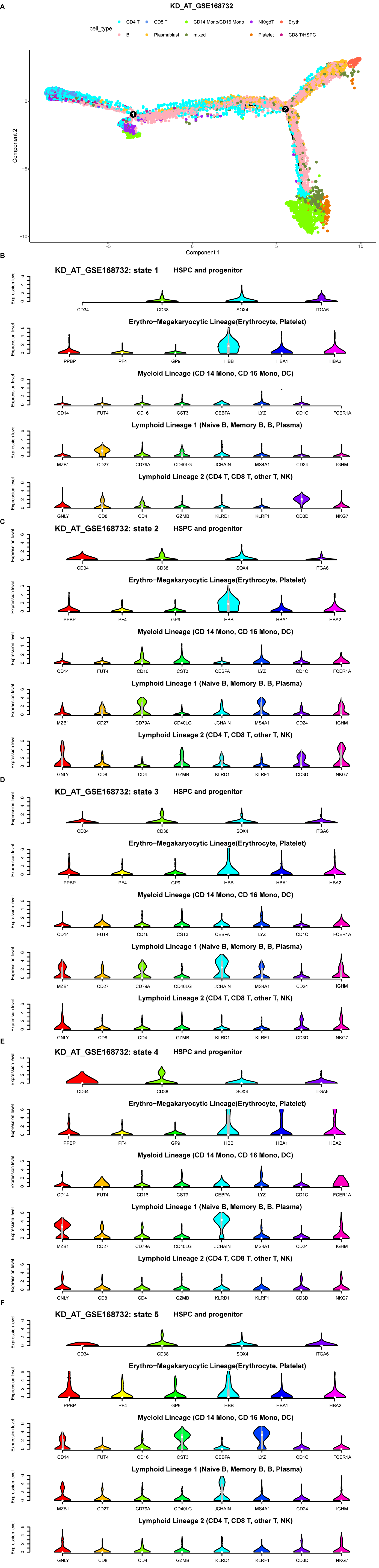

Supplement: Supplementary Figure S6 — Pseudo-time analysis of cell developmental trajectory in KD patients after treatment of GSE168732 dataset. (A) The differentiation trajectory of all cells in KD patients after treatment by cell types in GSE168732 dataset. (B) The canonical markers of five cell lineages for state 1 in KD patients after treatment in GSE168732 dataset. (C) The canonical markers of five cell lineages for state 2 in KD patients after treatment in GSE168732 dataset. (D) The canonical markers of five cell lineages for state 3 in KD patients after treatment in GSE168732 dataset. (E) The canonical markers of five cell lineages for state 4 in KD patients after treatment in GSE168732 dataset. (F) The canonical markers of five cell lineages for state 5 in KD patients after treatment in GSE168732 dataset. [file Image6.tif]

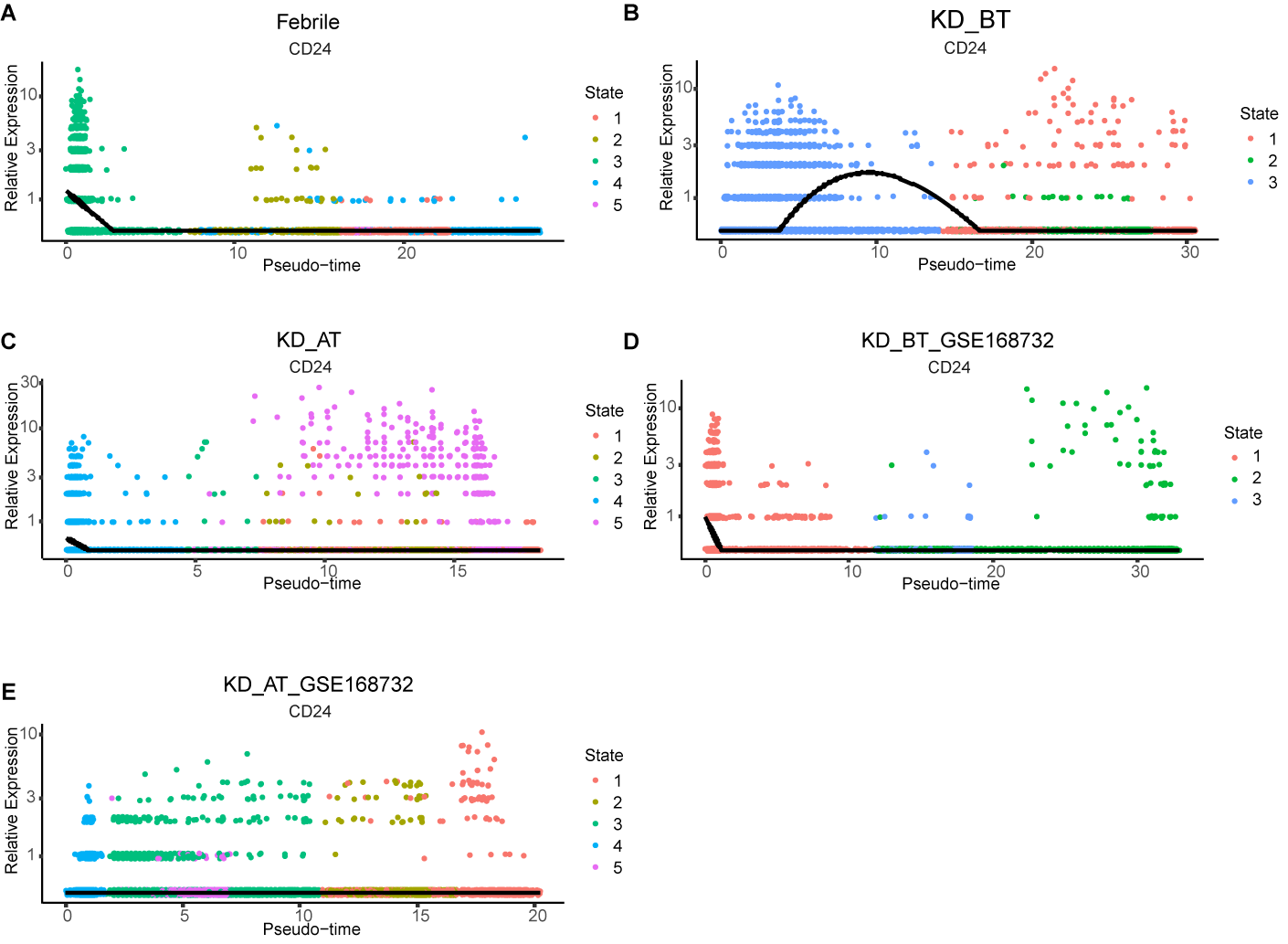

Supplement: Supplementary Figure S7 — Pseudo-time analysis of expression dynamics of CD24 in cell developmental trajectory in our dataset and GSE168732 dataset. (A) Expression dynamics of CD24 in febrile patients of our dataset. (B) Expression dynamics of CD24 in KD patients before treatment of our dataset. (C) Expression dynamics of CD24 in KD patients after treatment of our dataset. (D) Expression dynamics of CD24 in KD patients before treatment of GSE168732 dataset. (F) Expression dynamics of CD24 in KD patients after treatment of GSE168732 dataset. [file Image7.tif]

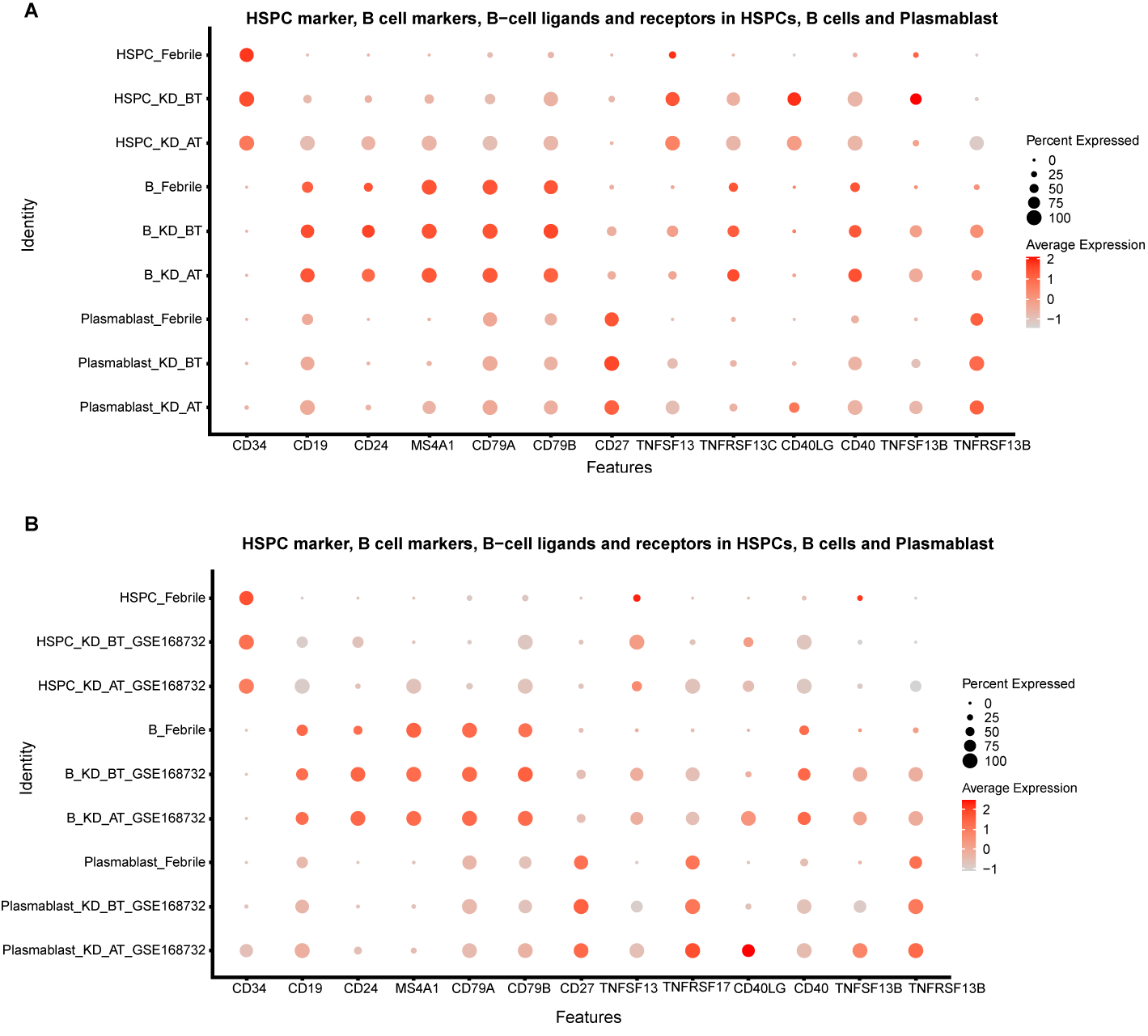

Supplement: Supplementary Figure S8 — Dot plot for HSPC marker, B cell markers, B-cell ligands and receptors in our dataset and GSE168732 dataset. (A) The expression of HSPC marker, B cell markers, B-cell ligands and receptors in HSPCs, B cells and plasma blast cells in our dataset. (B) The expression of HSPC marker, B cell markers, B-cell ligands and receptors in HSPCs, B cells and plasma blast cells in GSE168732 dataset. [file Image8.tif]

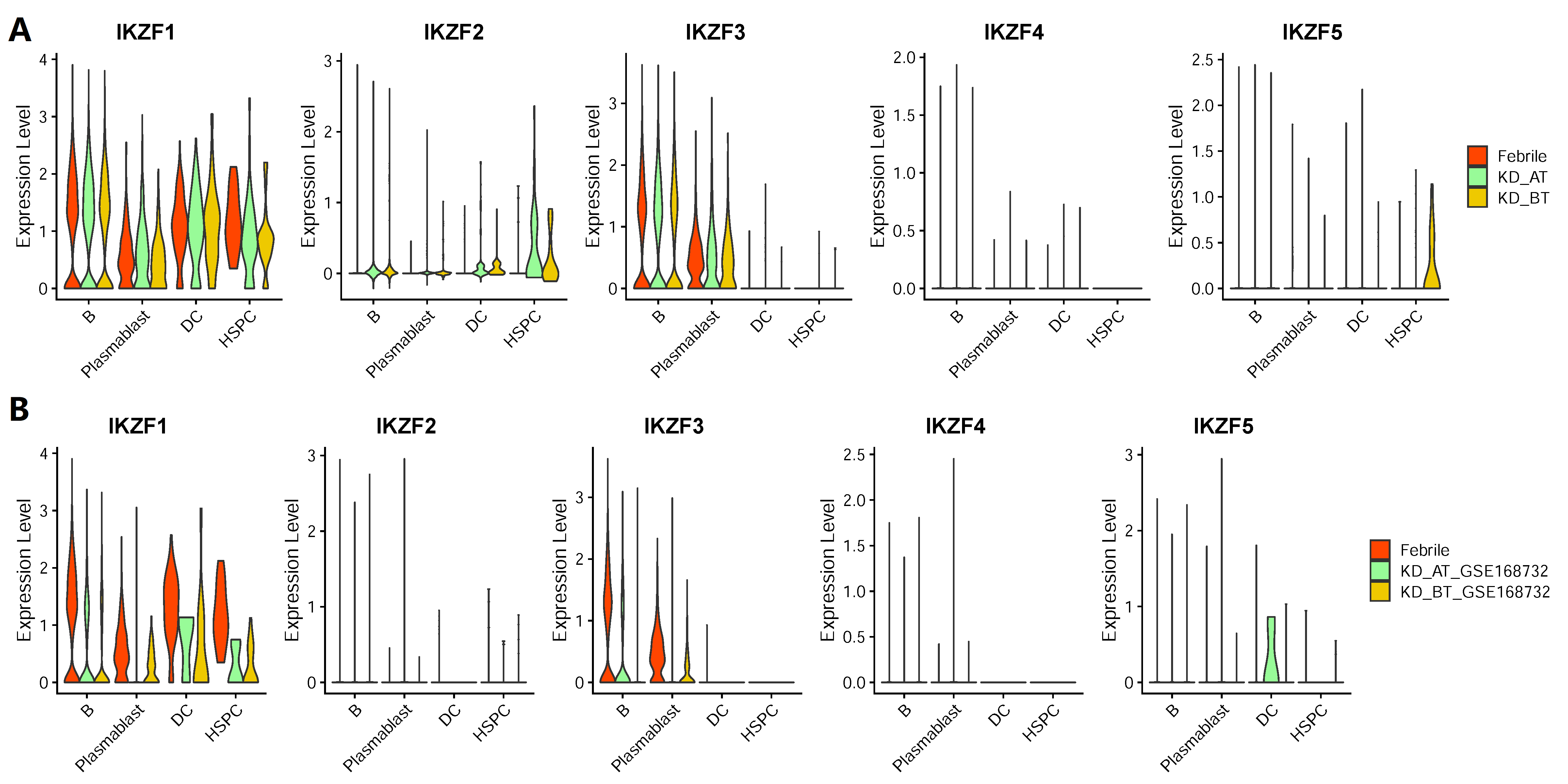

Supplement: Supplementary Figure S9 — Violin plot for five IKZF genes in our dataset and GSE168732 dataset. (A) The expression of five IKZF genes in HSPC, DC, plasma blast and B cells in our dataset. (B) The expression of five IKZF genes in HSPC, DC, plasma blast and B cells in GSE168732 dataset. [file Image9.tif]

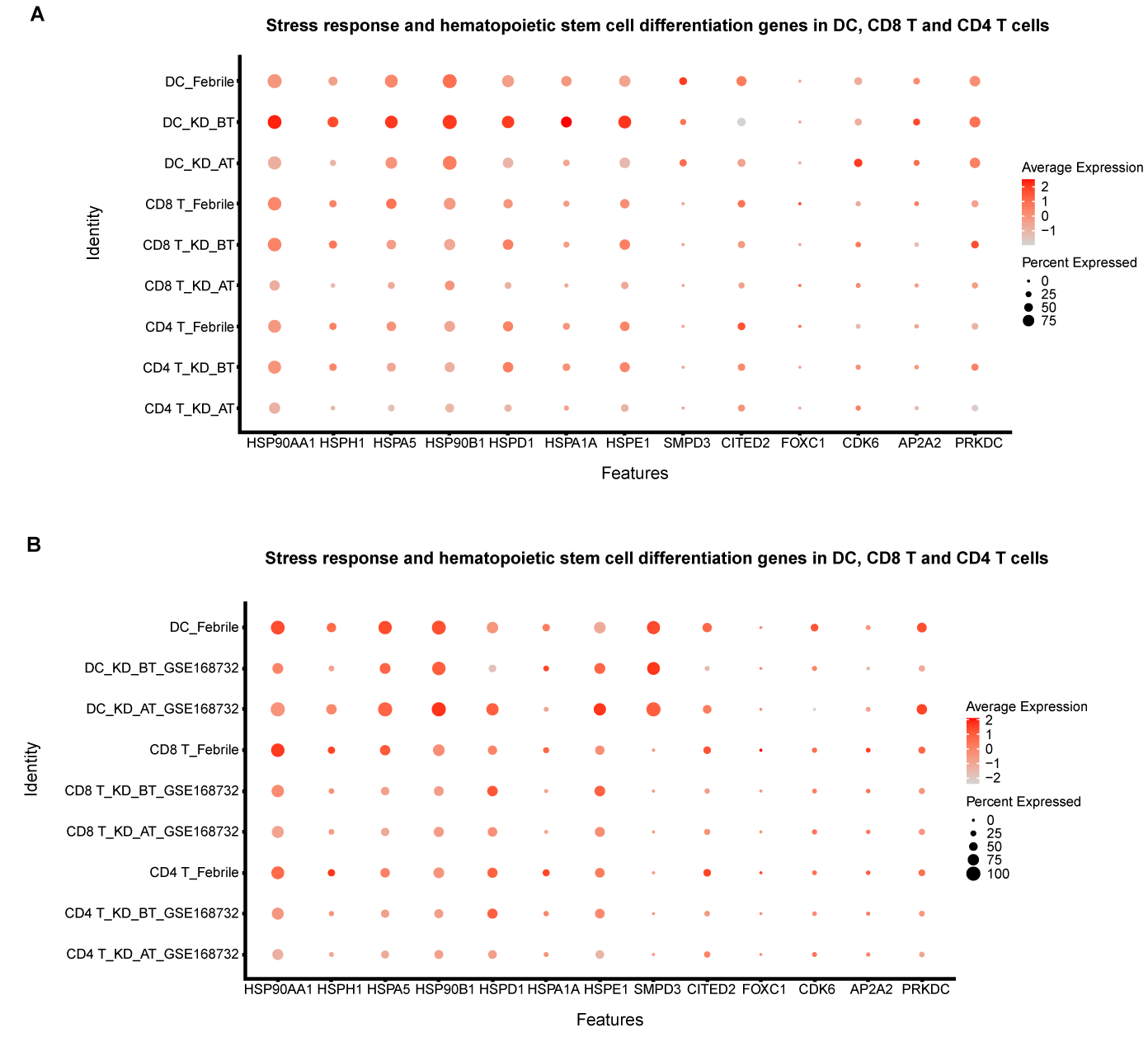

Supplement: Supplementary Figure S10 — Dot plot for stress response and hematopoietic stem cell differentiation genes in our dataset and GSE168732 dataset. (A) The expression of stress response and hematopoietic stem cell differentiation genes in DC, CD8 T and CD4 T cells in our dataset. (B) The expression of stress response and hematopoietic stem cell differentiation genes in DC, CD8 T and CD4 T cells in in GSE168732 dataset. [file Image10.tif]
